# Sepsis in obstetrics score: Predicting morbidity among antenatal and postnatal women

**DOI:** 10.6026/973206300212090

**Published:** 2025-07-31

**Authors:** Rubina Dohare, Somlina Roy, Avneesh Arya, Sachin Parmar

**Affiliations:** 1 Department of Obstetrics and Gynaecology, Government Medical College Datia, Madhya Pradesh, India; 2 Department of Obstetrics and Gynaecology, Amaltas Institute of Medical Science, Dewas, Madhya Pradesh, India; 3 Department of Surgery, Government Medical College, Datia, Madhya Pradesh, India; 4 Department of Community Medicine V.K.S. Government Medical College, Neemuch, Madhya Pradesh, India

**Keywords:** Maternal sepsis, sepsis in obstetrics score, pregnancy-associated infections, intensive care, maternal mortality, resource-limited settings

## Abstract

Maternal sepsis is a leading cause of morbidity and mortality worldwide, with a significant impact in resource-limited settings like India. The Sepsis in Obstetrics Score (SOS) has been validated as an effective tool to predict intensive care needs in obstetric sepsis. This prospective observational study, conducted at Gandhi Medical College, Bhopal, from January 2021 to June 2022, included 225 antenatal and postnatal women meeting two or more Systemic Inflammatory Response Syndrome (SIRS) criteria. The study found that higher SOS scores were associated with worse maternal and fetal outcomes, including increased maternal mortality and fetal complications like stillbirth. The SOS score proves to be a useful tool for early identification of high-risk obstetric patients, particularly in low-resource settings.

## Background:

Maternal sepsis is a serious health problem that can kill a mother and is still a major global public health concern. It is a major cause of illness and death in mothers, especially in both developed and developing countries. The World Health Organisation (WHO) says that puerperal sepsis is the third leading cause of maternal death worldwide, accounting for about 15% of the estimated 500,000 maternal deaths each year [1]. About 60% of women who die during childbirth or right after giving birth die [2]. Maternal sepsis is thought to affect 4.4% of women around the world and it is responsible for 10% of maternal deaths worldwide. But there isn't much data from low- and lower-middle-income countries, like India. A 2021 study looked at national and regional trends in maternal mortality in India and found that the main causes of maternal death were obstetric haemorrhage (47%), pregnancy-related infections (12%) and hypertensive disorders (7%), especially in states with low incomes [3]. So, infections that happen during pregnancy are the second most common cause of death during childbirth in the country. According to the International Consensus, sepsis is "life-threatening organ dysfunction resulting from a dysregulated host response to infection" [4]. Maternal sepsis is when a woman gets sepsis during pregnancy, childbirth, after an abortion, or in the weeks after giving birth, specifically from the start of labour or the breaking of membranes to 42 days after giving birth [5]. There are many distal, intermediate and proximal risk factors that contribute to its development. For example, changes in the immune system and body during pregnancy can make it harder to diagnose [6]. Streptococci, Staphylococci, Escherichia coli, Clostridium tetani, Clostridium welchii, Chlamydia and Gonococci are some of the bacteria that can cause puerperal sepsis. These germs can come from inside the body, from outside the body, or from a hospital. Some signs of puerperal sepsis are fever with chills, pain in the lower abdomen, foul-smelling lochia, a uterus that isn't fully developed, vaginal bleeding and, in severe cases, septic shock. Infections can move from the uterus to nearby pelvic organs and tissues, which can lead to parametritis, peritonitis, septicaemia and long-term problems like infertility and pelvic inflammatory disease [5]. Early diagnosis and supportive care are key to good management. Early detection is very important for avoiding problems and getting better results. In obstetric cases, sepsis often starts in the perineum, cervix, vagina, or uterus. Albright *et al.* [7] created the Sepsis in Obstetrics Score (SOS), a validated clinical tool that can help doctors figure out if pregnant or postpartum women with suspected sepsis need to go to the hospital for critical care. It includes things like temperature, systolic blood pressure, heart rate, respiratory rate, oxygen saturation, white blood cell count, percentage of immature neutrophils and lactate levels. The SOS score was very good at predicting which patients needed intensive care in the original validation study. It had an area under the curve of 0.92, a sensitivity of 88.9% and a specificity of 99.2% and a negative predictive value of 99.9% [8]. The United Nations' Sustainable Development Goals (SDGs) aim is a global maternal mortality ratio (MMR) of fewer than 70 deaths per 100,000 live births by 2030 [9]. The Government of India supports this goal. To reach this goal, we need to collect a lot of data, keep an eye on maternal mortality accurately and know the specific reasons for it in each area. But in India and other countries with high death rates, a lot of births and maternal deaths go unrecorded, which makes it harder to keep track of [10, 11]. Maternal sepsis is still not well studied, especially in low- and middle-income countries, even though it is a big problem. The increase in maternal sepsis over the past ten years is concerning because it puts a strain on the healthcare system and can be avoided. There haven't been many studies that look at how the SOS score works in the real world and most of the studies that have been done are retrospective and based in high-income countries [12]. Therefore, it is of interest to predict morbidity among antenatal and postnatal women using SOS.

## Materials and Methods:

This was a prospective observational study conducted in the Department of Obstetrics and Gynaecology, Sultania Zanana Hospital, Gandhi Medical College, Bhopal. The study period extended from January 2021 to June 2022.A total of 225 antenatal and postnatal women (within 42 days postpartum) who presented to the emergency department during the study period were enrolled. Inclusion required fulfilment of two or more Systemic Inflammatory Response Syndrome (SIRS) criteria at the time of admission.

## Inclusion criteria:

[1] Antenatal and postnatal women (within 42 days of delivery) fulfilling at least two SIRS criteria at the time of admission.

[2] Willingness to provide informed consent.

## Exclusion criteria:

[1] Patients who did not meet SIRS criteria.

[2] Women unwilling to provide consent.

Prior approval was obtained from the Institutional Ethics Committee (Certificate No: 87/IEC/2021). Eligible participants were provided with detailed information about the study in a language they could understand and written informed consent was obtained.

## All patients were screened using the following SIRS criteria at admission:

[1] Mean arterial pressure <65 mmHg

[2] Systolic blood pressure ≤90 mmHg

[3] Heart rate ≥110/min

[4] Respiratory rate ≥22/min

[5] Temperature ≥38°C or ≤36°C

[6] Leucocyte count ≥14,000/mm^3^ or ≤4,000/mm^3^

Women meeting ≥2 criteria were enrolled and assessed using a structured proforma to record demographic details, clinical history and risk factors, physical and obstetric findings. Investigations included hemoglobin level, total leukocyte count, percentage of immature neutrophils and venous lactic acid levels. The SOS score was calculated for each participant. Management details including ICU admission, duration of hospital stay, complications and maternal outcomes were documented. Maternal and fetal monitoring was performed according to institutional protocols. Outcomes were recorded in terms of maternal morbidity (ICU admission, MODS, duration of stay) and mortality, as well as fetal outcomes (live birth, birth weight, gestational age at delivery, stillbirth and intrauterine fetal demise).

## Operational definitions:

[1] Age: Recorded in completed years as of January 1, 2021.

[2] Socioeconomic status: Assessed using Modified BG Prasad's Socioeconomic Scale 2019 based on per capita income.

[3] Gravida and parity: Defined as per standard obstetric definitions.

[4] Gestational age: Calculated from the last menstrual period (LMP).

[5] Weight: Measured using a standardized weighing scale.

## Results:

Distribution of patients by sepsis score, case type, age, booking status and socio-economic status (n = 225) shows the breakdown of various patient demographics and characteristics based on their sepsis scores. It provides insights into the relationship between the sepsis score and case type, age group, booking status and socio-economic status of the patients. Table 2 (see PDF) illustrate Elements of the risk factors linked to sepsis score under a different category covered ante-partum period, intra-partum period, associated medical and obstetric conditions, factors at hospital stay, genital infections, breast conditions, and oro-dental hygiene. P-values and chi-square are given on each category. P-values lower than 0.05 are statistically significant. Distribution of patients according to risk factors (n = 225) provides an analysis of various risk factors such as gestational age, parity, antenatal care visits and other clinical factors during the antepartum, intrapartum and postpartum periods.

The Chi-square values and p-values indicate the significant associations between these risk factors and sepsis severity, particularly highlighting antepartum factors like gestational age and antenatal care visits. Clinical features and mode of delivery with sepsis in obstetric score (n = 225) outlines the clinical features associated with sepsis and the mode of delivery. It reports the frequency of different clinical features like fever, abdominal pain, dyspnea and wound infection and also examines the distribution of normal vaginal and cesarean deliveries across patients with varying sepsis scores. To properly cite the tables within the text, you can integrate the references to each table like this: Maternal and fetal outcomes (Table 4 - see PDF) present the maternal and fetal outcomes based on sepsis scores. It includes the rates of maternal discharge, maternal mortality, transfers and fetal outcomes, such as live birth, stillbirth and intrauterine fetal death (IUFD). Significant associations with outcomes like transfer and IUFD are highlighted with Chi-square values and p-values. Out of 225 patients, 38.2% had sepsis scores <6 and 61.8% had scores >6 (Table 1 - see PDF). In ANC and puerperal cases, 45.9% and 54.1% had lower scores, while 49.6% and 50.4% had higher scores. Most were 18-25 (43%), with older people having higher sepsis scores. Most patients (60%) were registered, but unbooked (8%) had more high scores. Lower socioeconomic groups had higher sepsis rates and 48% were upper lower class. These findings link higher sepsis scores to older age, unbooked status and lower socioeconomic status. In this cohort, preterm birth, high parity, inadequate antenatal care and repeated intrapartum interventions were significantly linked to higher sepsis scores. Medical conditions like hypertension, gestational diabetes, respiratory infections and UTIs also elevated risk. Conversely, factors like anemia, chorioamnionitis and delivery location were not statistically significant. These results highlight key contributors to sepsis vulnerability in obstetric patients. Among 225 patients; fever (53.6%) was the most common clinical feature, followed by wound infections (26.2%) and abdominal pain (19.8%) (Table 3 - see PDF). Most deliveries, both vaginal and LSCS, occurred at full term, though preterm births showed a higher sepsis burden. Overall, 61.8% had sepsis scores >6, with clinical signs predominantly involving fever and wound-related issues. In this cohort, higher sepsis scores were linked to worse maternal and fetal outcomes. Patients with scores >6 had increased transfer rates (6.8% vs. 1.7%), higher maternal mortality (1.8% vs. 0.6%) and significantly more fetal complications, including stillbirths and IUFD (p = 0.017) (Table 4 - see PDF). These results underscore the prognostic value of the sepsis in obstetric score.

## Discussion:

This study is observational studies that shows the current spread of maternal sepsis and demonstrates the usefulness of a Sepsis in Obstetrics Score (SOS) to risk stratify both antenatal and postnatal women in a low-resource environment. The prevalence of sepsis observed (1.04%) is also consistent with past findings which show that despite this being a fairly rare complication, sepsis is a substantial cause of maternal illness and fatalities especially in areas where healthcare facilities and data are scarce. The existing illness severity grading systems, typical of APACHE, SAPS, and SOFA, although useful in most ICU groups, fail to consider the peculiarities of the physiological compensation in pregnancy. This gap is closed with the help of SOS which was developed specifically with obstetric population to assess its risk through based on the certain parameters which are being added in relation to the specific physiological parameters of pregnancy. The age (27.8 6.6 years) of this present cohort was similar to the findings of Agrawal *et al.* [8] implying consistency on the demographic setting of same population. Importantly, the results of this study did not find any statistical relationship between age and SOS scores; however, according to the findings of Balki *et al.* [13], odds of severe sepsis were reported higher in women older than 35, which prove age as one of the risk factors in a particular setting. Though the socioeconomic status indicated no significant correlation, the high number of women with lower socioeconomic status reported high scores on SOS, probably indicating the inability of such women to obtain early antenatal care. Other obstetric predictors like a preterm birth, high parity and inadequate ante natal visits were found to be significant predisposing factors to high SOS scores. To provide one of the examples, SOS >6 was common in the majority of preterm births, as well as in women who had less than four antenatal visits, which served as one more indication that regular antenatal visits keep pregnant women and their infants safe. High parity was also associated with the risk of sepsis as indicated by other studies before. The unbooked status became the main predictor of a bigger SOS score, in which most of the unbooked women obtained the SOS outcome above the risk threshold level. It is consistent with preliminary studies, which classify unbooked status as a significant risk factor of sepsis [14]. The intrapartum events, such as repeated vaginal checks, lengthy labour, and premature rupture of the membranes (PROM), had a strong connection with increased SOS scores. Demisse *et al.* [15] have underlined the role of these factors in the risk of sepsis development, and our results direct to their importance in practice. Chorioamnionitis also showed an increase in SOS scores, cocontiguous with a study that shows a significantly greater probability of sepsis in the women with the condition. According to a study by Acousta *et al.* [16], there is increased chance of sepsis in diabetic women by 47 percentages. Home delivery and the overall length of stay in hospital were also among the other risk factors that were identified to be important, whereby sanitary home births provided the greatest risk that the subject might succumb to puerperal infection as also shown by Ali *et al.* [17].

Genital infections were common in high-SOS women and poor oral hygiene was noted in some of them but was not significant at a 95 per cent confidence level in this study. The highest presenting symptom was fever followed by wound infection and abdominal pain in line with the symptom situation described by Bakhtawar *et al.* [14]. As far as the outcomes of deliveries are concerned, there was a strong relationship between the mode of delivery and SOS score, and the cases of cesarean section (LSCS) represented the higher risks in line with the researchers by Shivananda *et al.* [18] and Kankuri *et al.* [19]. The occurrence of maternal deaths and severe morbidities like HELLP syndrome, ARDS, and ICU admission had a direct relationship with SOS: 9 out of 10 maternal deaths and serious morbidities were associated with SOS (6). This agrees with other findings reported by Agrawal *et al.* [8], which determined that pulmonary and multi-organ involvement were some of the main manifestations of severe sepsis. High maternal SOS scores had also adverse effects on fetal outcomes, whereby all intrauterine fetal demises and the majority of stillbirths were encountered in this group favoring the association between severe maternal sepsis and adverse perinatal outcomes as established by Balki *et al.* [13].

## Conclusion:

Sepsis is a leading and preventable cause of maternal mortality worldwide, highlighting the need for early detection tools. The SOS score proves effective in identifying high-risk obstetric patients with pregnancy-associated sepsis, helping prioritize care in emergency settings. This study underscores the SOS score's potential in differentiating severe from non-severe sepsis, emphasizing its utility in resource-limited countries for allocating critical care. Further validation of the SOS score in obstetric sepsis is essential.

## Funding:

The study received no external funding or sponsorship.

## References

[1] https://www.who.int/.

[2] https://pubmed.ncbi.nlm.nih.gov/21290641/.

[3] Bonet M (2017). Reprod Health..

[4] Singer M (2016). JAMA..

[5] https://www.nhp.gov.in/disease/gynaecology-and-obstetrics/puerperal-sepsis.

[6] Lämmle L (2013). Int J Environ Res Public Health..

[7] Albright C.M (2014). Obstet Gynecol..

[8] Agarwal R (2019). Obstet Med..

[9] https://www.who.int/reproductivehealth/.

[10] Jha P (2014). BMC Med..

[11] Gomes M (2017). Health Aff (Millwood)..

[12] Bauer M.E (2013). Anesth Analg..

[13] Balki I (2022). Can J Anaesth..

[14] Bakhtawar S (2020). BMC pregnancy and childbirth..

[15] Demisse G.A (2019). BMC pregnancy and childbirth..

[16] Acosta C.D (2016). BMJ open..

[17] Ali T.S (2006). J Pak Med Assoc..

[18] Shivananda R.P (2020). Int J Reprod Contracept Obstet Gynecol..

[19] Kankuri E (2003). Acta obstetricia et gynecologica Scandinavica..

